# An update on XMEN disease.

**DOI:** 10.1007/s10875-020-00790-x

**Published:** 2020-05-26

**Authors:** Juan C. Ravell, Samuel D. Chauvin, Tingyan He, Michael Lenardo

**Affiliations:** 1 Laboratory of Clinical Immunology and Microbiology, Division of Intramural Research (DIR), National Institute of Allergy and Infectious Diseases (NIAID), Bethesda, MD, USA;; 2 Molecular Development of the Immune System Section, Laboratory of Immune System Biology, and Clinical Genomics Program, DIR, NIAID, Bethesda, MD, USA;; 3 Department of Rheumatology and Immunology, Shenzhen Children’s Hospital, Shenzhen 518038, China.

## Abstract

“X-linked immunodeficiency with magnesium defect, Epstein-Barr virus (EBV) infection, and neoplasia” (XMEN) disease is an inborn error of glycosylation and immunity caused by loss of function mutations in the magnesium transporter 1 (*MAGT1*) gene. It is a multisystem disease that strongly affects certain immune cells. MAGT1 is now confirmed as a non-catalytic subunit of the oligosaccharyltransferase complex and facilitates Asparagine (N)-linked glycosylation of specific substrates, making XMEN a congenital disorder of glycosylation manifesting as a combined immune deficiency. The clinical disease has variable expressivity and impaired glycosylation of key MAGT1-dependent glycoproteins in addition to Mg^2+^ abnormalities can explain some of the immune manifestations. NKG2D, an activating receptor critical for cytotoxic function against EBV, is poorly glycosylated and invariably decreased on CD8^+^ T cells and natural killer (NK) cells from XMEN patients. It is the best biomarker of the disease. The characterization of EBV-naïve XMEN patients has clarified features of the genetic disease that were previously attributed to EBV infection. Extra-immune manifestations, including hepatic and neurological abnormalities have recently been reported. EBV-associated lymphomas remain the main cause of severe morbidity. Unfortunately, treatment options to address the underlying mechanism of disease remain limited and Mg^2+^ supplementation has not proven successful. Here, we review the expanding clinical phenotype and recent advances in glycobiology that have increased our understanding of XMEN disease. We also propose updating XMEN to “**X**-linked **M**AGT1 deficiency with increased susceptibility to **E**BV-infection and **N**-linked glycosylation defect” in light of these novel findings.

## Introduction:

Variable expressivity frequently poses a challenge when trying to generalize the clinical features of rare diseases, but elucidation of the molecular etiology of congenital diseases allows clinicians to tie together variable phenotypes to a common disease pathway. “X-linked immunodeficiency with magnesium defect, Epstein-Barr virus (EBV) infection, and neoplasia” (XMEN) disease is a combined immunodeficiency (CID) caused by hemizygous loss of function (LOF) mutations in the magnesium transporter 1 gene (*MAGT1*) located on the X chromosome [1–4]. Additional cases have recently been reported, expanding the clinical phenotype and shedding light on previously unrecognized features of the disease[4–12]. Advances in glycobiology have revealed that MAGT1 deficiency is a selective congenital disorder of glycosylation (CDG) that predominantly affects the immune system[4, 13]. Herein, we review the most up-to-date scientific and clinical evidence of this fascinating yet complex disease. We also propose updating the XMEN acronym to “**X**-linked **M**AGT1 deficiency with increased susceptibility to **E**BV-infection and **N**-linked glycosylation defect” to better reflect these new findings.

## Epidemiology and genetics

A total of 36 unique male patients have been reported [2–8, 10–12, 14, 15]. Females with heterozygous *MAGT1* mutations are healthy carriers with a pattern of X chromosome inactivation (XCI) skewed towards the normal allele in their hematopoietic cells [1, 11, 12]. The disease appears to have complete penetrance with variable expressivity. *MAGT1* has 10 exons, is located on chromosome Xq21.1, and predicted to have multiple in-frame translation initiation sites with the predominant form encoding a 335 amino acid (AA) protein (UniProtKB-Q9H0U3). Most deleterious *MAGT1* variants abolish protein expression (Table 1) [4].

## MAGT1: a putative magnesium transporter.

Mg^2+^ is the second most abundant intracellular cation after potassium and is essential for numerous biological functions [16]. *MAGT1* was first identified as a gene transcript upregulated in mouse kidney cells cultured in low magnesium [17]. Electrophysiological studies using patch clamping revealed that expression of human MAGT1 in *Xenopus* oocytes mediates a very specific Mg^2+^ uptake[17]. However, MAGT1 has little similarity to any known cation transporter and rather shares a 66% AA sequence homology with the human Tumor Suppressor Candidate 3 protein (TUSC3), a member of the oligosaccharyl transferase (OST) complex responsible for the asparagine (N)-linked glycosylation (NLG)[18, 19]. MAGT1 and TUSC3 are the human homolog of the yeast OST subunits OST3/6[20]. Both proteins can rescue the growth arrest of the *Saccharomyces cerevisiae* mutant strain *alr1*Δ, a yeast lacking the ALR1 Mg^2+^ transporter, which only proliferate at high concentrations of extracellular magnesium, suggesting overlapping functions between MAGT1 and TUSC3[18]. Similarly, MAGT1 partially rescues survival and proliferation of a chicken B-cell line (DT40) lacking the Mg^2+^ channel “Transient receptor potential cation channel subfamily M member 7” (TRPM7)[21]. Early studies on XMEN patients suggested that loss of MAGT1 resulted in a decreased free intracellular Mg^2+^ concentration [Mg^2+^] and impaired T-cell receptor (TCR)-induced transient Mg^2+^ influx, but the specificity of the fluorescent Mg^2+^ indicators has been questioned [1, 2]. The molecular relationship to OST subunits and complexities of Mg^2+^ investigation suggested that key manifestations of disease could revolve around defective glycosylation in addition to or instead of alterations of Mg^2+^ transport.

## MAGT1: a subunit of the OST complex.

**Figure 1:**
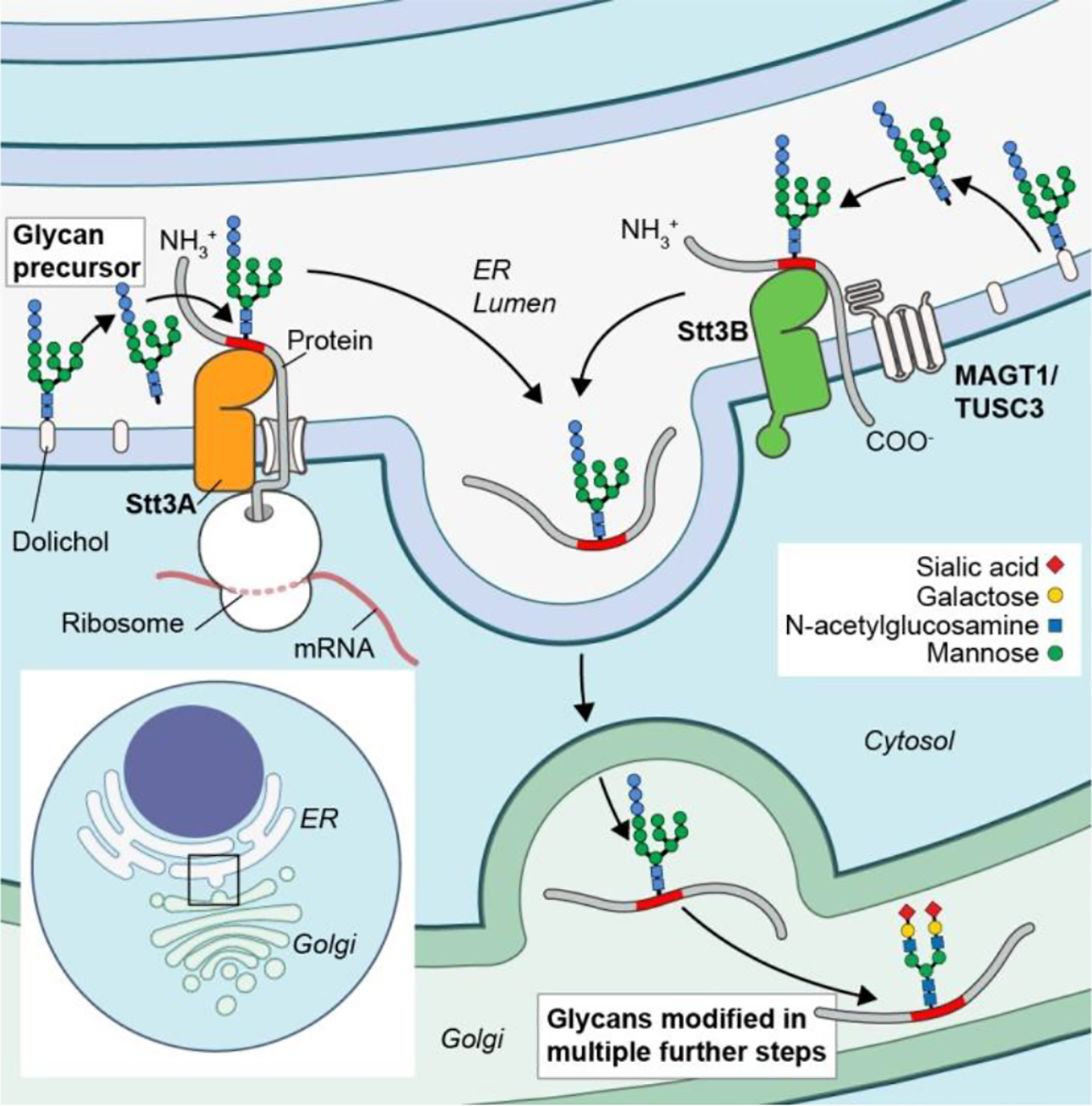
MAGT1 is a facilitator of N-linked glycosylation. A dolichol-linked glycan precursor is assembled from monosaccharides units and en block transferred to proteins in the endoplasmic reticulum (ER) by either STT3A or STT3B, which are the catalytic subunits of the oligosaccharyltransferase (OST) complex. Either MAGT1 or TUSC3 associate with the STT3B-containing OST complex. Other accessory subunits common to both STT3A- and STT3B-OST complexes are not shown for simplicity. STT3A and STT3B catalyze the transfer of the pre-assembled glycans co-translationally and post-translationally, respectively. The transferred glycan is then further processed and modified in the ER and Golgi apparatus.

Protein glycosylation is an essential co-translational and post-translational process by which carbohydrates (glycans) are covalently attached to proteins, including at least 50% of the human proteome[22]. Glycans play an essential role in the maturation, stability, localization, and function of glycoproteins, a large number of which play critical roles in normal immune function[23]. In humans, the predominant form of protein glycosylation is NLG, a multistep process that covalently attaches glycans to asparagine residues in proteins. First, a lipid-linked oligosaccharide (LLO) is synthesized from monosaccharide units in the cytosol and ER. This LLO is then transferred to the nascent protein in the ER. Finally, the glycan is processed and further modified in the ER and Golgi apparatus (Figure 1) [24–27]. Although early studies placed it as a plasma membrane protein, it was more recently revealed that MAGT1 mainly localizes in the endoplasmic reticulum and is a non-catalytic subunit of the OST complex[13, 18, 28]. Each OST complex contains several accessory subunits, non-covalently associated to an enzymatic subunit, either STT3A or STT3B, which catalyzes the transfer of the pre-assembled glycans from the LLO onto asparagine residues of nascent polypeptides with the AA sequence “Asparagine-X-Serine or Threonine” (N-X-S/T), where X is any amino acid except proline [24, 28–30] (Figure 1). STT3A functions co-translationally, whereas STT3B acts post-translationally and glycosylates sites that were skipped by STT3A[31–33]. Either MAGT1 or TUSC3 can associate with the STT3B-OST complex in a mutually exclusive way and facilitate the NLG of a subset of STT3B-dependent glycoproteins (Figure 1)[28]. Both MAGT1 and TUSC3 have a thioredoxin (TRX) domain containing a bi-cysteine motif (CXXC) and four transmembrane (TM) regions[13, 18, 19]. Although described as necessary for disulfide-mediated cross-linking to certain STT3B-dependent peptide substrates, CXXC deletion did not impair MAGT1-dependent glycosylation in a tumor cell line model system[13, 28]. Importantly, expression of TUSC3 in cells devoid of MAGT1 is able to rescue the glycosylation defect of MAGT1-dependent glycoproteins, which demonstrates functional redundancy between MAGT1 and TUSC3[13, 28]. This compensatory capacity suggests that the clinical phenotype resulting from loss of either MAGT1 or TUSC3 could mainly depend on their differential tissue expression. We have shown that immune cells do not express TUSC3 and exclusively rely on MAGT1 to facilitate NLG of specific STT3B-dependent glycoproteins, which explains why XMEN patients have a predominantly immunological phenotype[13]. However, a few other tissues, including the liver, predominantly express MAGT1 over TUSC3, potentially explaining some of the non-immune findings like liver disease[13].

## XMEN disease: the biochemistry of a selective CDG.

**Figure 2:**
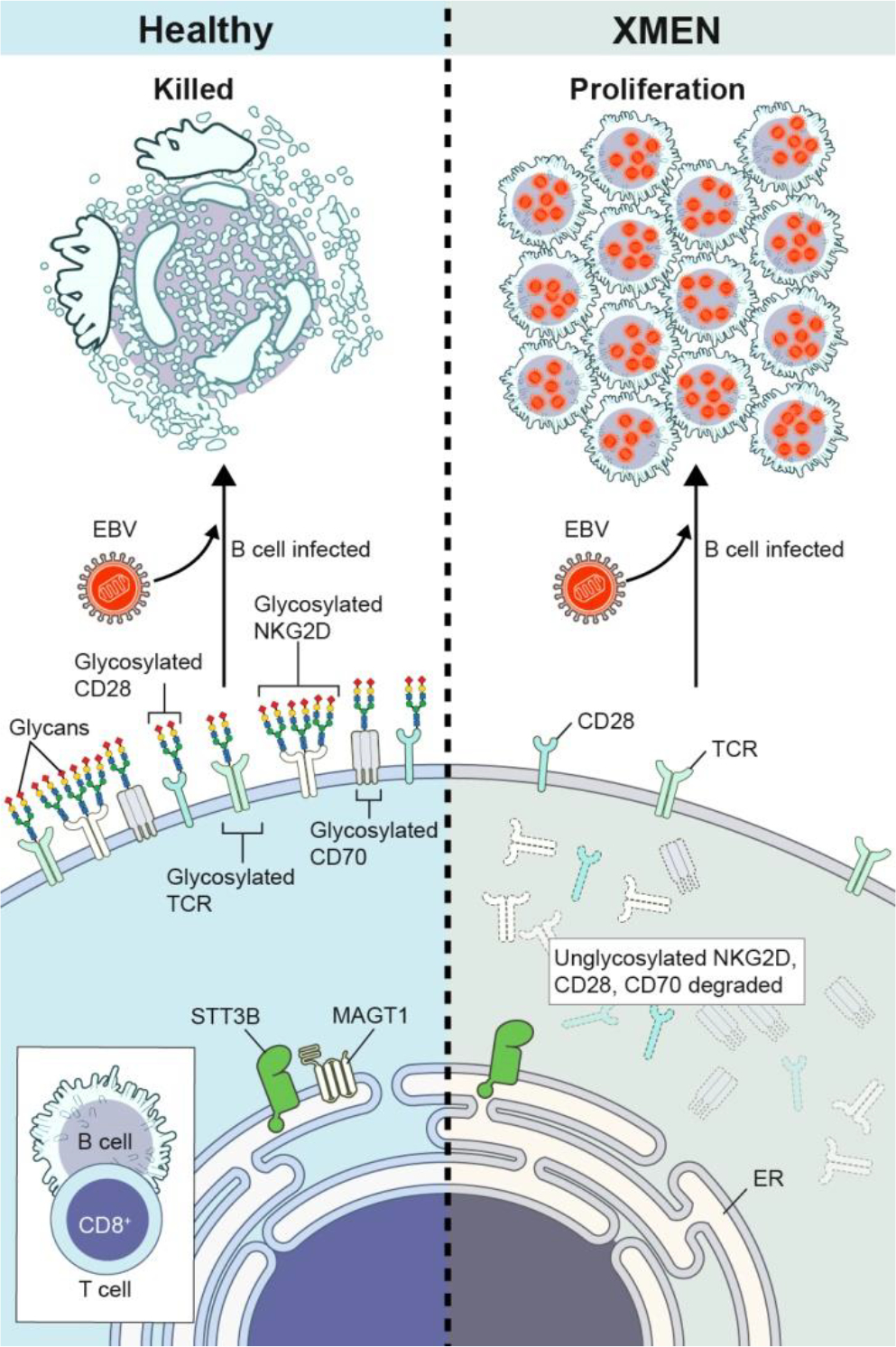
Defective glycosylation of key immune molecules and pathogenesis of XMEN disease. Immune cells, including CD8^+^ T lymphocytes, do not express TUSC3 and rely exclusively on MAGT1 to facilitate the N-linked glycosylation of some specific STT3B-substrates. Loss of MAGT1 in XMEN lymphocytes results in underglycosylation of key immune molecules, including NKG2D, CD28, CD70, and the T-cell receptor (TCR). In healthy individuals, these fully glycosylated proteins are normally expressed. In XMEN disease, decreased glycosylation of some molecules leads to their degradation resulting in their loss or decreased surface expression as is the case for NKG2D, CD28, and CD70. Other glycoproteins, such as the TCR, although hypoglycosylated in XMEN disease, are still expressed on the cell surface. Loss of NKG2D and CD70 expression impairs the cytotoxic activity of lymphocytes against EBV-infected B cells and result in increased risk of EBV-associated lymphoproliferation and lymphoma in XMEN disease.

Congenital disorders of glycosylation (CDG) are a group of more than 130 monogenic diseases that cause abnormal glycosylation[34, 35]. While their clinical manifestations and severity are variable, neurodevelopmental abnormalities, intellectual disability (ID), failure to thrive and liver disease are commonly observed [36–38]. Analysis of serum transferrin glycosylation status (carbohydrate deficient transferrin, CDT), has historically been used as a screening and classification tool for CDG that affect NLG. Transferrin is mainly produced by hepatocytes and has two N-linked glycosylation sites with two negatively charged sialic acids each [39]. Defects that affect the assembly or transfer of the glycan produce a Type-I CDT pattern, and those that affect their downstream processing a Type-II pattern[40, 41]. XMEN patients have a Type-I CDT pattern with minor defects in final processing (Type-II), supporting the role of MAGT1 as a facilitator of NLG[4, 11]. Furthermore, *in vitro* studies using fibroblasts from patients with deleterious *MAGT1* variants revealed a glycosylation defect in only STT3B-dependent glycoproteins[11]. Interestingly, impaired glycosylation was more pronounced in cells from a patient that expressed a mutant MAGT1 protein compared with those with absent MAGT1 expression[11]. An important link between immune deficiency and NLG in XMEN became apparent with our observation that the decreased expression of the activator receptor “Natural-Killer Group 2, member D” (NKG2D) and other important immune molecules was due to impaired glycosylation [4]. Because NK and CD8^+^ T cells do not express TUSC3, loss of MAGT1 results in a partially glycosylated NKG2D protein that is degraded in cells from XMEN patients[4]. Quantitative glycoproteomic analysis revealed that surprisingly few glycoproteins are differentially glycosylated in lymphocytes from XMEN patients [4]. Similar to studies in the yeast OST3/6 knock out, the affected peptides in XMEN had an inverted NXS/NXT ratio, showing that MAGT1 preferentially facilitates glycosylation of peptides with NXS motifs[4, 42, 43]. While most of the affected glycosites in XMEN were predicted to be STT3B-dependent, a small proportion were STT3A-dependent, and most (62 %) were within 60 amino acids of a TM domain[4]. These results demonstrate the importance of MAGT1 in NLG and hint at a model in which MAGT1 helps the OST complexes target glycosites near a TM region. The differentially glycosylated peptides in XMEN lymphocytes mapped to 73 unique genes that encode proteins important for immunity, neural function, glycosylation, transport, adhesion, immunity, and lipid metabolism[4]. Because the glycoproteomics analysis was only carried out in T lymphocytes, it is likely that there is a broader range of intracellular proteins in different cell types with defective glycosylation in the absence of MAGT1. In lymphocytes, defective glycosylation was observed for key immune proteins including the T cell receptor α and β chains (TCR-α/β), the costimulatory molecules CD28 and CD70, the major histocompatibility complex protein HLADRB1, the ceramide synthase 2 (CERS2) and the Solute Carrier Family 4 Member 7 (SLC4A7) proteins[4]. Hypoglycosylation correlated with decreased surface expression for some (NKG2D, CD28, CD70 and HLA-DRB) but not all (TCR-α/β) glycoproteins (Figure 2)[4]. Although most differentially glycosylated proteins had decreased glycosylation compared with healthy controls, a few glycoproteins were hyperglycosylated[4]. Transfection of lymphocytes from XMEN patients with *MAGT1* mRNA rescued the glycosylation defects confirming the direct role of MAGT1 as facilitator of NLG[4]. Additional glycoproteomic analyses in saliva and plasma also revealed defective glycosylation in XMEN[13]. In plasma, the Ig heavy chain, haptoglobin, and hemoglobin showed decreased glycosylation; while in saliva, the heavy chain of IgA, lactoperoxidase, prolactin-inducible protein, and haptoglobin were hypoglycosylated in XMEN[13]. Transcriptome analysis of CD8^+^ T cells done to assess the effect of defective glycosylation in gene expression patterns, showed that the CD28 pathway was strongly inhibited in XMEN[13]. This is consistent with the reduced glycosylation and decreased surface expression of CD28 in XMEN cells[4, 13]. Mg^2+^ is an essential cofactor for many enzymes, and lymphocytes from healthy individuals cultured in Mg^2+^-deprived media have decreased glycosylation and surface expression of MAGT1-dependent glycoproteins[13]. *In vitro* and *in vivo* studies on a few XMEN patients showed that Mg^2+^ supplementation partially recovered NKG2D expression, which translated to better cytotoxic function against EBV, but lack of response has also been reported [2, 12]. Unfortunately, Mg^2+^ supplementation does not appear to be a useful and effective clinical intervention in XMEN disease as originally hypothesized[2, 12, 44]. Recent discoveries support the critical role of MAGT1 in NLG and a key role for this pathway in disease pathogenesis, but we don’t yet understand how defective NLG and Mg^2+^ transport are related in this disease.

## Clinical manifestations

**Figure 3:**
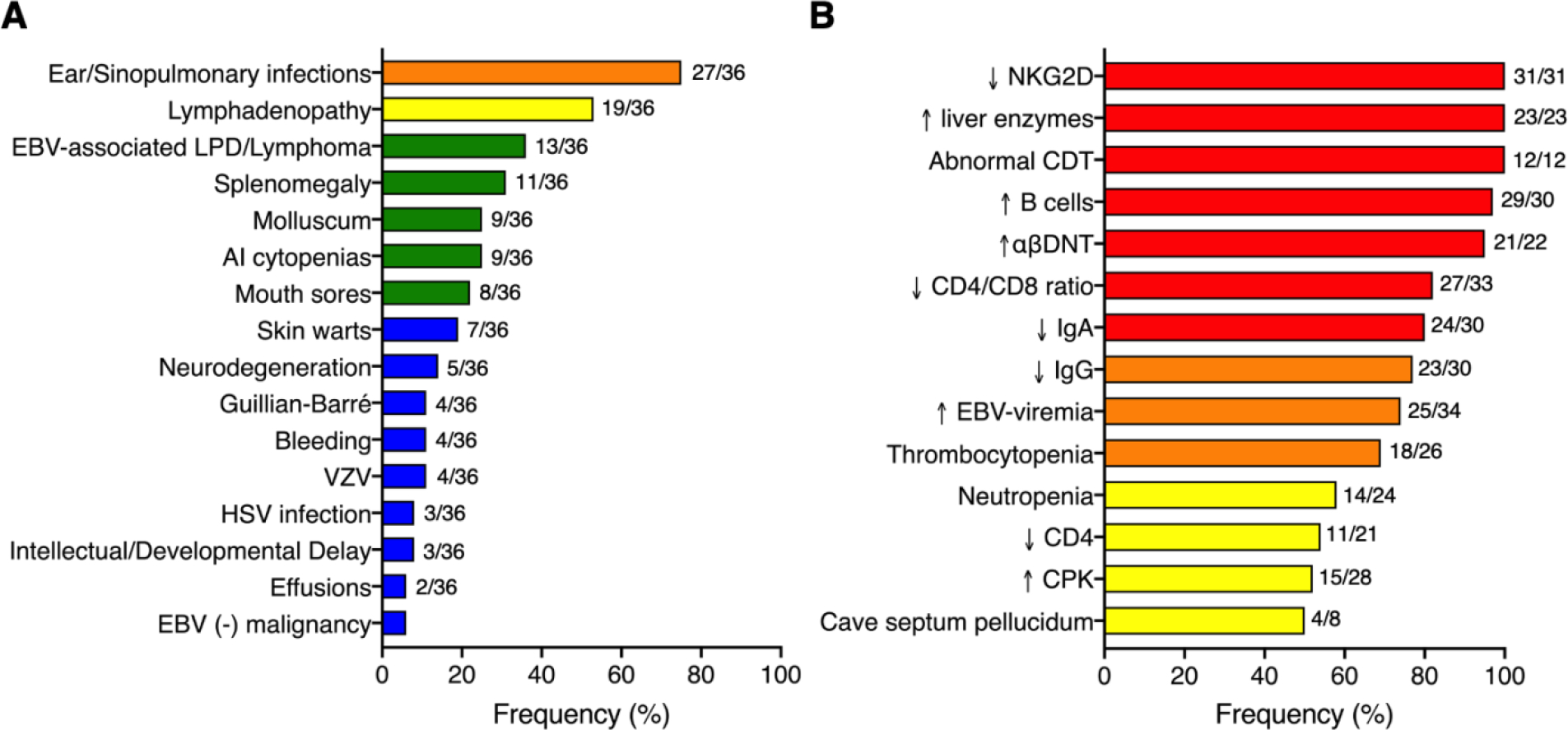
Clinical and laboratory findings in XMEN disease. The percentage of all XMEN patients who presented with the given clinical manifestations (**A**) and laboratory findings (**B**). For clinical manifestations, the percentage of all reported patients with each phenotype is given. For the laboratory findings, only the percentage of patients who have had testing reported are shown. The number of patients reported with each symptom out of the number of patients tested is given for each. Bars are color coded for the prevalence of that finding in XMEN disease; red is 80–100 %, orange is 60–79 %, yellow is 40–59 %, green is 20–39 %, and blue is ≤ 19 %.

The most common clinical features in XMEN are summarized in Figure 3, and a more thorough system-by-system discussion follows.

## Immunodeficiency and immune dysregulation

XMEN disease is a CID characterized by increased susceptibility to chronic EBV infection and EBV-associated lymphoproliferation, sinopulmonary and ear infections, lymphadenopathy (LAD), dysgammaglobulinemia, and autoimmune cytopenias. XMEN may have a more indolent course than other combined immunodeficiencies (CID) with some patients not receiving a diagnosis until adulthood[4, 45]. EBV is a widely disseminated oncogenic B cell-tropic γ−herpesvirus that infects some children and the majority of adults worldwide[46–49]. The identification of EBV-naïve patients revealed that the immune phenotype of EBV-naïve and EBV-infected patients is very similar[4]. Invariably, all patients have decreased NKG2D expression on both natural killer (NK) and CD8^+^ T cells, making it the best biomarker and a hallmark of the disease [4]. Most patients have decreased serum IgG and IgA with impaired response to polysaccharide antigens, and frequent ear and sinopulmonary infections, sometimes associated with bronchiectasis[4]. Similar to *Magt1*-knockout mice, XMEN patients often have elevated B cells with a significant expansion of the naïve subset[4, 50]. Low CD4/CD8 ratio and elevated CD4^−^CD8^−^TCRαβ^+^ T (αβDNT) cells are commonly observed, while recent thymic emigrants, NK and natural killer T (NKT) cell counts are usually normal[4]. Almost half of the patients have CD4 T cell lymphopenia, but life-threatening opportunistic infections are rare in the absence of concomitant immunosuppressive treatment[4]. An infant with severe pneumonia had *Pneumocystis jirovecii* and cytomegalovirus in the bronchoalveolar lavage[15]. Progressive multifocal leukoencephalopathy (PML) was reported in one XMEN patient during chemotherapy for an underlying lymphoma[5]. Severe molluscum contagiosum occurs in about one-third of the patients, while skin warts are less frequently observed[4]. Condyloma accuminatum requiring surgical excision was reported in one young adult[4]. Varicella zoster virus (VZV) infection and herpes simplex virus (HSV) infections have also been reported[3, 12, 15]. Mild, sometimes transient, thrombocytopenia is a common hematological abnormality, even in patients with no splenomegaly and no history of autoimmune (AI) cytopenias[4]. Transient neutropenia with or without associated mouth sores is present in more than half of the patients[4]. A leukemoid reaction was reported in an EBV-naïve infant with no clonal lymphoproliferative disorder[4]. A significant hemorrhagic risk, usually disproportionate for the degree of thrombocytopenia and involving mucosal sites, has been identified in some patients[10, 15]. This bleeding risk appears to be a relatively common and severe complication after allogenic bone marrow transplant (BMT) in XMEN, for which a precise mechanism has not been identified[10]. Autoimmune hemolytic anemia (AIHA) and/or immune thrombocytopenia (ITP) occur in about one third of the patients and have been the presenting symptom in some EBV-naïve patients[4]. Lymphocytes from XMEN patients have decreased calcium flux and phospholipase C-gamma 1 (PLCγ1) activation, and impaired re-stimulation induced cell death upon TCR crosslinking, likely due to impaired signaling through the hypoglycosylated TCR and CD28[1, 4]. Interestingly, *Magt1*-knockout mice show no defects in their T cell activation, but have hyper-activation of their B cells[50]. Unlike humans, mice express TUSC3 in their immune cells, which may account for some of these differences[51]. Both CD8^+^ T cells and NK cells of XMEN patients have impaired EBV-specific cytotoxic function *in vitro*, consistent with the persistent EBV viremia in most patients and decreased glycosylation of NKG2D and CD70 (Figure 2) [2, 4].

## Non-malignant lymphoproliferation

Prior to the identification of EBV-naïve patients, it was thought that the observed lymphadenopathy (LAD) in XMEN was primarily due to EBV-infection. However, we recently reported that LAD was also frequently seen in EBV-naïve patients while splenomegaly was mostly observed in about 50% of the EBV-infected patients[4]. In our NIH cohort, most patients have mild LAD involving the cervical, axillary, intra-abdominal, and/or inguinofemoral lymph nodes bilaterally. Histopathological examination of lymph node (LN) biopsies from two EBV-naïve children were consistent with Castleman disease, with an atypical B cell population co-expressing the CD5 marker identified in one of these cases[4, 12]. Reactive lymphoid hyperplasia has been observed in LN biopsies from both EBV-naïve and EBV-infected patients[4]. Follicular hyperplasia with or without atypical lymphoid proliferation, and increased number of EBV-positive cells have been reported in LN, tonsillar and adenoidal tissue biopsies from EBV-infected individuals[4]. Histopathology of the terminal ileum of an EBV-infected patient showed reactive mucosal lymphoid follicles[4]. An atypical perivascular EBV^+^ lymphoproliferative cutaneous lesion was also recently reported in a young adult with a history of multiple EBV-associated lymphomas[12].

## Malignancies

Twelve patients have developed either lymphoma or EBV-positive lymphoproliferative disease (EBV^+^LPD) (Table 2). Classical Hodgkin lymphoma (CHL) is the most common malignancy in XMEN patients. EBV-positive LPD, Burkitt’s lymphoma, and diffuse large B-cell lymphoma (DLBCL) have also been reported. The age at diagnosis ranges from 7 to 57 years, with most cases occurring in the second and third decades of life. Five patients experienced more than one malignancy. Few EBV-negative cancers have been reported. A 27-year-old male with a history of CHL who was successfully treated with chemo- and radio- therapy subsequently developed an EBER-negative liposarcoma[4, 10]. One child with CD4-lymphopenia developed human herpes virus 8 (HHV-8)-positive Kaposi sarcoma (KS)[6]. Although EBV has been implicated in the pathogenesis of other malignancies, including nasopharyngeal carcinoma (NPC) and gastric cancer, these have not been observed in XMEN.

## Liver disease

Asymptomatic transient elevations in aspartate aminotransferase (AST) and alanine aminotransferase (ALT) are common in XMEN and were observed in all of our patients[4]. Although some patients have sustained transaminase elevations, most patients have fluctuations in their liver enzymes, including determinations within the normal range. This is true for both EBV-naïve and EBV-infected patients. None of the patients in our NIH cohort had impaired hepatic function, gamma-glutamyl transpeptidase (GGT) levels were mostly normal, and hepatitis serologies were all negative[4]. Although autoimmune hepatitis (AH) has been reported for one case, we did not find any serologic or histopathological evidence of AH in a larger cohort of patients [4, 45]. Varying degrees of inflammation, fibrosis, hepatosteatosis, iron deposition, and diffuse glycogenosis were seen in both EBV-infected and EBV-naïve patients[4]. Hepatic biopsies from patients with EBV viremia did not show a pattern of injury consistent with EBV-associated hepatitis and were Epstein-Barr encoding region (EBER) negative[4]. Taken together, these findings suggest that the liver abnormalities in XMEN are not related to EBV-infection and may be secondary to the underlying NLG defect [4].

## Neurological and cognitive abnormalities

The association of MAGT1 with intellectual disability (ID) has been a matter of controversy for years. Pathogenic variants in *TUSC3* have been linked to autosomal recessive intellectual disability and developmental delay[52–54]. The identification of a *MAGT1* variant that co-segregated with ID in a family with 5 siblings suggested that deleterious variants in *MAGT1* could be a cause of X-linked ID[54]. However, this variant (later referred to as c.1028C>T, pV343G) was subsequently found to be relatively common in the general population and highly unlikely to be disease-causing[55]. Recently, a boy with the *MAGT1* missense mutation c.1068A>C, p.K356N presented with intellectual and developmental disability[11]. In contrast to all other reported pathogenic *MAGT1* genetic alterations that result in complete loss of protein expression, this variant encoded a mutant protein with normal expression[11]. The authors hypothesized that expression of this non-functional protein could prevent incorporation of TUSC3 in the STT3B-OST complex[11]. Because the brain expresses TUSC3, this could account for the ID observed in this patient though NKG2D expression (not reported) would be needed for further functional validation. Another child with a c.991C>T, p.R331X *MAGT1* mutation, which results in complete loss of protein expression, was also described to have intellectual and developmental disability[11]. However, two other patients from unrelated families and an immunodeficiency phenotype had the same *MAGT1* mutation but no ID[4]. Cognitive and language delay was also described in a child with immunodeficiency and a large *MAGT1* gene deletion extending to the proximity of the *ATRX* promoter, a gene associated with ID[6]. In a large cohort of XMEN patients with LOF *MAGT1* mutations resulting in complete loss of protein expression, ID was not observed[4]. These findings suggest that complete loss of MAGT1 alone is unlikely to cause ID whereas expression of non-functional *MAGT1* mutants may contribute to ID.

We recently reported a 30-year-old patient with a pathogenic variant in *MAGT1* (c.414C>A, p.Y138X) and no history of malignancy who developed progressive neurological decline with marked brain, cerebellar, and spinal cord atrophy as a young adult[4]. Although the presenting symptoms were neurological, the immune phenotype was consistent with XMEN[4]. This prompted us to further investigate the prevalence of central nervous system (CNS) abnormalities in XMEN. In our NIH cohort, 7 additional patients had brain imaging done by either magnetic resonance imaging (MRI) or computerized tomography (CT) scans. Two patients in their fifth decade of life had brain atrophy greater than expected for age, and two patients had white matter abnormalities consistent with leukoencephalopathy but all of them had received chemotherapy for treatment of an underlying EBV-associated malignancy[4]. A 52-year-old patient with MRI findings suggestive of posterior reversible encephalopathic syndrome (PRES) secondary to chemotherapy for EBV^+^LPD, had persistence of the brain MRI changes and associated atrophy 4 years later[5]. A 10-year old boy had strokes secondary to right middle cerebral artery vasculitis[12]. Three NIH patients have had seizures[4]. Acute immune-mediated polyneuropathy or Guillain-Barre syndrome (GBS) has been reported in 4 patients, including an EBV-naïve individual[4, 7]. Interestingly, half of the patients with brain imaging in our cohort had cavum septum pellucidum (CSP), a cerebrospinal fluid (CSF)-filled space between the leaflets of the septum pellucidum that is normally present until 3–6 months of age and is only found in approximately 15% of the adult population[4]. These findings suggest that neurological abnormalities in XMEN may be more prevalent than previously recognized. Finally, asymptomatic elevations in creatine phosphokinase (CPK), sometimes exacerbated by exercise, have also been observed[4].

## Diagnosis and treatment

XMEN disease should be suspected in male patients with recurrent ear and sinopulmonary infections, LAD with or without splenomegaly, chronic EBV-infection, EBV-associated lymphoproliferative disease, and autoimmunity. A positive family history of immunodeficiency or lymphoma in maternal male relatives reinforces the clinical suspicion. Although most patients have persistent EBV viremia after infection, a growing number of EBV-naïve patients, mostly young children, have been identified, and the absence of EBV viremia does not rule out the disease. Determination of NKG2D expression on CD16^+^CD56^+^ NK cells and/or CD8^+^ T cells by flow cytometry should be done in all patients suspected of having XMEN. The CDT test, although not specific, is a widely available clinical test that could be used in places where NKG2D expression determination is not available or to reinforce the diagnosis of XMEN disease. Conversely, male patients with a suspected CDG, including mild ID and/or liver disease, and a CDT isoform analysis most consistent with a mild type I pattern should be evaluated for XMEN disease. Although rare, it seems prudent to consider XMEN disease in the differential diagnosis of male patients with neurodegeneration, especially if associated with immune deficiency or EBV viremia. Both ionized and total serum magnesium concentrations are usually normal and play no role in the diagnosis of XMEN disease. Notably, CD70 deficiency and some malignancies may also decrease NKG2D expression[56, 57]. Therefore, molecular testing to identify genetic alterations in *MAGT1* should be performed when the clinical suspicion is high and to confirm the diagnosis in patients with decreased NKG2D expression.

A growing number of inborn errors of immunity have been associated with increased predisposition to EBV-associated LPD and should be considered in the differential diagnosis. These include SH2D1A (SAP), XIAP, ITK, CD27, CD70, NFκB1, CTPS1, RASGRP1, and CORO1A deficiencies, which are reviewed elsewhere[58, 59]. The combination of LAD, AI cytopenias, and elevated αβDNT present in a subset of XMEN patients resembles autoimmune lymphoproliferative syndrome (ALPS), a disease of defective FAS-mediated apoptosis[4, 7, 60]. We recently showed that, in contrast to ALPS, XMEN patients do not have the commonly observed serum elevations in FasL, IL-10, IL-18, and vitamin B_12_; and XMEN αβDNT cells do not have increased expression of the CD45R (B220) marker as do their ALPS counterparts[4].

Management of XMEN patients should be individualized based on clinical manifestations. Most patients have mild disease and live a normal lifestyle. We routinely check EBV serologies to assess past infection, and EBV viral load by polymerase chain reaction (PCR) in whole blood to determine and follow-up viremia. A thorough initial evaluation aimed at establishing baseline disease status is an important part in the management of XMEN disease. In addition to assessing LAD and/or splenomegaly, we advocate for a thorough initial pulmonary, hepatic, and neurological evaluation. Prophylactic antibiotics and Ig replacement therapy significantly improve ear and sinopulmonary infections. We approach the AI cytopenias in XMEN similarly to their ALPS counterparts and advise against splenectomy[61]. We do not recommend anti-CD20 therapy with rituximab for EBV control in the absence of EBV-associated B cell malignancy. While rituximab can deplete B cells from peripheral blood and lower the viral load, it does not eliminate B cells from the tissues. Recurrent treatment with rituximab in other immune deficiencies has sometimes selected for CD20^−^EBV^+^ B cells, which could give raise to B-cell malignancies not amenable to anti-CD20 therapy[62]. Furthermore, rituximab has been associated with some cases of PML in primary immunodeficiencies, including XMEN[5, 63]. The identification of EBV-naïve patients raises multiple questions about preventive strategies for this population. There is no FDA-approved EBV vaccine though candidates are currently under investigation. Ig replacement and antivirals do not prevent EBV infection. The role of magnesium supplementation is currently being investigated in a clinical trial[64]. Gene therapy is not available yet. Allogenic BMT has been successful in a few patients, but the post-transplant mortality remains high[10, 12]. It is also unclear whether restoration of normal immune function alone may prevent the non-immune manifestations.

## Conclusions

XMEN is a monogenic disease caused by deleterious molecular alteration in *MAGT1* and has a variable clinical phenotype. Several recent studies have increased our understanding of the disease manifestations and pathogenesis. With the identification of EBV-naïve patients, we learned that there is an underlying cellular and clinical phenotype that is not just a response to chronic EBV-infection. The larger number of reported cases have revealed that XMEN is a multisystem disease. The recognition of MAGT1 as a facilitator of NLG has provided a new perspective of XMEN as a CDG that predominantly manifests as a CID. However, the precise mechanism by which MAGT1 is involved in the homeostasis of Mg^2+^ and how this affects the glycosylation defect requires further investigation. We look forward to future work aimed at restoring the NLG defect in this complex disease.

## Figures and Tables

**Table 1:** Reported deleterious *MAGT1* molecular alterations

Mutation		Confirmation		References
cDNA	Protein	WB	NKG2D	
c.97A>T	p.M33L	N/A	↓*	[12]
c.110G>A	p.W37X	Undetectable	↓	[3, 4]
c.223C>T	p.Q75X	Undetectable	↓	[4, 15]
c.236G>A	p.W79X	Undetectable	↓	[4]
c.409C>T	p.R137X	Undetectable	↓	[3, 4, 10]
c.414C>A	p.Y138X	Undetectable	↓	[4]
c.472del	p.D158MfsX6	N/A	↓	[8]
c.555dup	p.Y186IfsX2	Undetectable	↓	[4, 7]
c.598delC	p.R200GfsX13	Undetectable	↓	[3, 4]
c.712C>T	p.R238X	Undetectable	↓	[4, 5, 10]
c.737_738insGA	p.F246LfsX18	N/A	↓	[15]
c.771T>A	p.C257X	N/A	↓*	[12]
c.774delT	p.F258LfsX5	Undetectable	↓	[4]
c.859_997del139	p.N287X	Undetectable	↓	[3, 4]
c.901_902insAA	p.T301KfsX14	Undetectable	↓	[4, 10]
c.938T>G	p.L313X	Undetectable	↓	[11]
c.991C>T	p.R331X	Undetectable	↓	[4, 11]
c.1068A>C	p.K356N	Normal	N/A	[11]
Partial gene deletion introns 1–8	N/A	Undetectable	↓	[4]
Partial gene deletion exons 2–10	N/A	Undetectable	↓	[6]
Partial gene deletion exons 3–10	N/A	Undetectable	↓	[4]

WB: detection of MAGT1 protein by immunoblotting. NKG2D: surface expression of NKG2D on CD8^+^ T cells and/or NK cells by flow cytometry. N/A: data not available.

* No direct comparison to healthy controls.

**Table 2:** Cancer Predisposition in XMEN disease

**ID**	**Age at cancer diagnosis**	**Currentage**	**Type**	**EBV viremia**	**EBER (tumor)**	**Outcome**
B.1[3]	45	45†	Lymphoma	+	N/A	Deceased after HSCT
D.1[3, 10]	12	20†	EBV-positive LPD of the hypothalamus*	+	+	CR (7 years), deceased after HSCT
E.1[3]	7, 13	22	Burkitt’s lymphoma	+	+	CR (9 years)
F.1[3]	17, 22	23†	Classical Hodgkin lymphoma	+	N/A	Deceased after HSCT
I.2[10]	13	31	EBV-positive LPD of the palate*	+	+	CR, Alive after HSCT (>1 year)
	27		Liposarcoma		-	
I.1[5]	52	58†	EBV-positive LPD	+	N/A	Deceased after chemotherapy
	57		Diffuse large B-cell lymphoma			
K.2	15	15	Classical Hodgkin lymphoma	+	+	In treatment
M.2[10]	16	18	EBV positive LPD*	+	+	Alive after HSCT
						(>1 year)
N.1	29	49	Classical Hodgkin lymphoma	+	N/A	CR (20 years)
R1 [7]	15	18†	Classical Hodgkin lymphoma	+	N/A	CR (3 years), deceased after HSCT
[6]	5	N/A	Kaposi sarcoma	+	N/A	CR at age 6^‡^
	9		Classical Hodgkin lymphoma		N/A	
[12]	21	31	Classical Hodgkin lymphoma	+	+^§^	Alive
	26		Burkitt’s lymphoma		+^§^	
[15]	15	15	Classical Hodgkin lymphoma	+	N/A	Alive

ID: patient identifier. Patients listed with a letter followed by a number have been previously reported by our group[4]. N/A not available. HSCT: allogeneic hematopoietic stem cell transplantation. CR: complete remission. Number of years in CR or after HSCT is shown in parenthesis.

* Although no lymphoma was detected, given the severity of the disease EBV-positive LPD is included in this table.

† Age at death.

§ Reported as “EBV positivity.”

‡ Patients clinical condition at the time of publication.

## References

[1] Li FY, , Second messenger role for Mg2+ revealed by human T-cell immunodeficiency. Nature, 2011 475(7357): p. 471–6. 21796205 10.1038/nature10246 PMC3159560

[2] Chaigne-Delalande B, , Mg2+ regulates cytotoxic functions of NK and CD8 T cells in chronic EBV infection through NKG2D. Science, 2013 341(6142): p. 186–91. 23846901 10.1126/science.1240094 PMC3894782

[3] Li FY, , XMEN disease: a new primary immunodeficiency affecting Mg2+ regulation of immunity against Epstein-Barr virus. Blood, 2014 123(14): p. 2148–52. 24550228 10.1182/blood-2013-11-538686 PMC3975255

[4] Ravell JC, , Defective glycosylation and multisystem abnormalities characterize the primary immunodeficiency XMEN disease. J Clin Invest, 2020 130(1): p. 507–522. 31714901 10.1172/JCI131116 PMC6934229

[5] Dhalla F, , Identification of a novel mutation in MAGT1 and progressive multifocal leucoencephalopathy in a 58-year-old man with XMEN disease. J Clin Immunol, 2015 35(2): p. 112–8. 25504528 10.1007/s10875-014-0116-2 PMC6328310

[6] Brigida I, , Large Deletion of MAGT1 Gene in a Patient with Classic Kaposi Sarcoma, CD4 Lymphopenia, and EBV Infection. J Clin Immunol, 2017 37(1): p. 32–35. 27770395 10.1007/s10875-016-0341-y PMC5226982

[7] Patiroglu T, , A case of XMEN syndrome presented with severe auto-immune disorders mimicking autoimmune lymphoproliferative disease. Clin Immunol, 2015 159(1): p. 58–62. 25956530 10.1016/j.clim.2015.04.015

[8] He TY, , [X-linked immunodeficiency with magnesium defect, Epstein-Barr virus infection, and neoplasia: report of a family and literature review]. Zhonghua Er Ke Za Zhi, 2018 56(1): p. 48–52. 10.3760/cma.j.issn.0578-1310.2018.01.013 29342998

[9] Akar HH, , Combined immunodeficiencies: twenty years experience from a single center in Turkey. Cent Eur J Immunol, 2016 41(1): p. 107–15. 27095930 10.5114/ceji.2015.56168 PMC4829808

[10] Dimitrova D, , Successful Bone Marrow Transplantation for XMEN: Hemorrhagic Risk Uncovered. J Clin Immunol, 2019 39(1): p. 1–3. 10.1007/s10875-018-0573-0 30470981

[11] Blommaert E, , Mutations in MAGT1 lead to a glycosylation disorder with a variable phenotype. Proc Natl Acad Sci U S A, 2019 116(20): p. 9865–9870. 31036665 10.1073/pnas.1817815116 PMC6525510

[12] Klinken EM, , Diversity of XMEN Disease: Description of 2 Novel Variants and Analysis of the Lymphocyte Phenotype. J Clin Immunol, 2019. 10.1007/s10875-019-00732-2 31865525

[13] Matsuda-Lennikov M, , Magnesium transporter 1 (MAGT1) deficiency causes selective defects in N-linked glycosylation and expression of immune-response genes. J Biol Chem, 2019. 10.1074/jbc.RA119.008903 PMC6746436 31337704

[14] Li FY, Lenardo MJ, and Chaigne-Delalande B, Loss of MAGT1 abrogates the Mg2+ flux required for T cell signaling and leads to a novel human primary immunodeficiency. Magnes Res, 2011 24(3): p. S109–14. 21983175 10.1684/mrh.2011.0286 PMC3732466

[15] Hoyos-Bachiloglu R, , The Many Faces of XMEN Disease, Report of Two Patients with Novel Mutations. J Clin Immunol, 2020. 10.1007/s10875-020-00746-1 31993868

[16] Romani A, Regulation of magnesium homeostasis and transport in mammalian cells. Arch Biochem Biophys, 2007 458(1): p. 90–102. 16949548 10.1016/j.abb.2006.07.012

[17] Goytain A and Quamme GA, Identification and characterization of a novel mammalian Mg2+ transporter with channel-like properties. BMC Genomics, 2005 6: p. 48. 15804357 10.1186/1471-2164-6-48 PMC1129089

[18] Zhou H and Clapham DE, Mammalian MagT1 and TUSC3 are required for cellular magnesium uptake and vertebrate embryonic development. Proc Natl Acad Sci U S A, 2009 106(37): p. 15750–5. 19717468 10.1073/pnas.0908332106 PMC2732712

[19] Mohorko E, , Structural basis of substrate specificity of human oligosaccharyl transferase subunit N33/Tusc3 and its role in regulating protein N-glycosylation. Structure, 2014 22(4): p. 590–601. 24685145 10.1016/j.str.2014.02.013

[20] Schulz BL, , Oxidoreductase activity of oligosaccharyltransferase subunits Ost3p and Ost6p defines site-specific glycosylation efficiency. Proc Natl Acad Sci U S A, 2009 106(27): p. 11061–6. 19549845 10.1073/pnas.0812515106 PMC2708779

[21] Deason-Towne F, Perraud AL, and Schmitz C, The Mg2+ transporter MagT1 partially rescues cell growth and Mg2+ uptake in cells lacking the channel-kinase TRPM7. FEBS Lett, 2011 585(14): p. 2275–8. 21627970 10.1016/j.febslet.2011.05.052 PMC3139019

[22] Apweiler R, Hermjakob H, and Sharon N, On the frequency of protein glycosylation, as deduced from analysis of the SWISS-PROT database. Biochim Biophys Acta, 1999 1473(1): p. 4–8. 10580125 10.1016/s0304-4165(99)00165-8

[23] Pascoal C, , CDG and immune response: From bedside to bench and back. J Inherit Metab Dis, 2019. 10.1002/jimd.12126 31095764

[24] Kornfeld R and Kornfeld S, ASSEMBLY OF ASPARAGINE-LINKED OLIGOSACCHARIDES. Annual Review of Biochemistry, 1985 54(1): p. 631–664. 10.1146/annurev.bi.54.070185.003215 3896128

[25] Schwarz F and Aebi M, Mechanisms and principles of N-linked protein glycosylation. Current Opinion in Structural Biology, 2011 21(5): p. 576–582. 21978957 10.1016/j.sbi.2011.08.005

[26] Shrimal S, Cherepanova NA, and Gilmore R, Cotranslational and posttranslocational N-glycosylation of proteins in the endoplasmic reticulum. Semin Cell Dev Biol, 2015 41: p. 71–8. 25460543 10.1016/j.semcdb.2014.11.005 PMC4442082

[27] Cherepanova N, Shrimal S, and Gilmore R, N-linked glycosylation and homeostasis of the endoplasmic reticulum. Curr Opin Cell Biol, 2016 41: p. 57–65. 27085638 10.1016/j.ceb.2016.03.021 PMC4983500

[28] Cherepanova NA, Shrimal S, and Gilmore R, Oxidoreductase activity is necessary for N-glycosylation of cysteine-proximal acceptor sites in glycoproteins. J Cell Biol, 2014 206(4): p. 525–39. 25135935 10.1083/jcb.201404083 PMC4137057

[29] Cherepanova NA, , Quantitative glycoproteomics reveals new classes of STT3A- and STT3B-dependent N-glycosylation sites. The Journal of Cell Biology, 2019 218(8): p. 2782–2796. 31296534 10.1083/jcb.201904004 PMC6683751

[30] Kelleher DJ, , Oligosaccharyltransferase isoforms that contain different catalytic STT3 subunits have distinct enzymatic properties. Mol Cell, 2003 12(1): p. 101–11. 12887896 10.1016/s1097-2765(03)00243-0

[31] Chen W, , Cotranslational folding and calnexin binding during glycoprotein synthesis. Proceedings of the National Academy of Sciences, 1995 92(14): p. 6229. 10.1073/pnas.92.14.6229 PMC41491 7541532

[32] Whitley P, Nilsson I, and von Heijne G, A Nascent Secretory Protein 5 Traverse the Ribosome/Endoplasmic Reticulum Translocase Complex as an Extended Chain. Journal of Biological Chemistry, 1996 271(11): p. 6241–6244. 8626416 10.1074/jbc.271.11.6241

[33] Shrimal S, Cherepanova NA, and Gilmore R, Cotranslational and posttranslocational N-glycosylation of proteins in the endoplasmic reticulum. Seminars in Cell & Developmental Biology, 2015 41: p. 71–78. 25460543 10.1016/j.semcdb.2014.11.005 PMC4442082

[34] Ng BG and Freeze HH, Perspectives on Glycosylation and Its Congenital Disorders. Trends Genet, 2018 34(6): p. 466–476. 29606283 10.1016/j.tig.2018.03.002 PMC5959770

[35] Francisco R, , The challenge of CDG diagnosis. Mol Genet Metab, 2019 126(1): p. 1–5. 30454869 10.1016/j.ymgme.2018.11.003

[36] Al Teneiji A, , Phenotypic and genotypic spectrum of congenital disorders of glycosylation type I and type II. Mol Genet Metab, 2017 120(3): p. 235–242. 28122681 10.1016/j.ymgme.2016.12.014

[37] Jaeken J and Matthijs G, Congenital disorders of glycosylation: a rapidly expanding disease family. Annu Rev Genomics Hum Genet, 2007 8: p. 261–78. 17506657 10.1146/annurev.genom.8.080706.092327

[38] Freeze HH, Congenital Disorders of Glycosylation: CDG-I, CDG-II, and beyond. Curr Mol Med, 2007 7(4): p. 389–96. 17584079 10.2174/156652407780831548

[39] Tegtmeyer LC, , Multiple phenotypes in phosphoglucomutase 1 deficiency. N Engl J Med, 2014 370(6): p. 533–42. 24499211 10.1056/NEJMoa1206605 PMC4373661

[40] Grünewald S, Matthijs G, and Jaeken J, Congenital Disorders of Glycosylation: A Review. Pediatric Research, 2002 52(5): p. 618–624. 12409504 10.1203/00006450-200211000-00003

[41] Majri SS, , STAT5B: A Differential Regulator of the Life and Death of CD4(+) Effector Memory T Cells. J Immunol, 2018 200(1): p. 110–118. 29187589 10.4049/jimmunol.1701133 PMC5736408

[42] Jamaluddin MF, Bailey UM, and Schulz BL, Oligosaccharyltransferase subunits bind polypeptide substrate to locally enhance N-glycosylation. Mol Cell Proteomics, 2014 13(12): p. 3286–93. 25118247 10.1074/mcp.M114.041178 PMC4256483

[43] Karaoglu D, Kelleher DJ, and Gilmore R, Functional characterization of Ost3p. Loss of the 34-kD subunit of the Saccharomyces cerevisiae oligosaccharyltransferase results in biased underglycosylation of acceptor substrates. J Cell Biol, 1995 130(3): p. 567–77. 7622558 10.1083/jcb.130.3.567 PMC2120544

[44] Ravell J and Lenardo M, manuscript in preparation. National Institute of Allergy and Infectious Diseases, National Institutes of Health.

[45] Ravell J, Chaigne-Delalande B, and Lenardo M, X-linked immunodeficiency with magnesium defect, Epstein-Barr virus infection, and neoplasia disease: a combined immune deficiency with magnesium defect. Curr Opin Pediatr, 2014 26(6): p. 713–9. 25313976 10.1097/MOP.0000000000000156 PMC4306042

[46] Cohen JI, Epstein-Barr virus infection. N Engl J Med, 2000 343(7): p. 481–92. 10944566 10.1056/NEJM200008173430707

[47] Balfour HH Jr., , Behavioral, virologic, and immunologic factors associated with acquisition and severity of primary Epstein-Barr virus infection in university students. J Infect Dis, 2013 207(1): p. 80–8. 23100562 10.1093/infdis/jis646 PMC3523797

[48] Dowd JB, , Seroprevalence of Epstein-Barr virus infection in U.S. children ages 6–19, 2003–2010. PLoS One, 2013 8(5): p. e64921. 23717674 10.1371/journal.pone.0064921 PMC3661547

[49] Condon LM, , Age-specific prevalence of Epstein-Barr virus infection among Minnesota children: effects of race/ethnicity and family environment. Clin Infect Dis, 2014 59(4): p. 501–8. 24820696 10.1093/cid/ciu342

[50] Gotru SK, , Cutting Edge: Imbalanced Cation Homeostasis in MAGT1-Deficient B Cells Dysregulates B Cell Development and Signaling in Mice. The Journal of Immunology, 2018 200(8): p. 2529. 29581357 10.4049/jimmunol.1701467

[51] Immuno-Navigator. Available from: https://genomics.virus.kyoto-u.ac.jp/immuno-navigator/.

[52] Garshasbi M, , A novel nonsense mutation in TUSC3 is responsible for nonsyndromic autosomal recessive mental retardation in a consanguineous Iranian family. Am J Med Genet A, 2011 155A(8): p. 1976–80. 21739581 10.1002/ajmg.a.34077

[53] Garshasbi M, , A defect in the TUSC3 gene is associated with autosomal recessive mental retardation. Am J Hum Genet, 2008 82(5): p. 1158–64. 18452889 10.1016/j.ajhg.2008.03.018 PMC2651624

[54] Molinari F, , Oligosaccharyltransferase-subunit mutations in nonsyndromic mental retardation. Am J Hum Genet, 2008 82(5): p. 1150–7. 18455129 10.1016/j.ajhg.2008.03.021 PMC2427205

[55] Piton A, Redin C, and Mandel JL, XLID-causing mutations and associated genes challenged in light of data from large-scale human exome sequencing. Am J Hum Genet, 2013 93(2): p. 368–83. 23871722 10.1016/j.ajhg.2013.06.013 PMC3738825

[56] Groh V, , Tumour-derived soluble MIC ligands impair expression of NKG2D and T-cell activation. Nature, 2002 419(6908): p. 734–8. 12384702 10.1038/nature01112

[57] Abolhassani H, , Combined immunodeficiency and Epstein-Barr virus-induced B cell malignancy in humans with inherited CD70 deficiency. J Exp Med, 2017 214(1): p. 91–106. 28011864 10.1084/jem.20160849 PMC5206499

[58] Latour S and Winter S, Inherited Immunodeficiencies With High Predisposition to Epstein-Barr Virus-Driven Lymphoproliferative Diseases. Front Immunol, 2018 9: p. 1103. 29942301 10.3389/fimmu.2018.01103 PMC6004768

[59] Tangye SG, Palendira U, and Edwards ES, Human immunity against EBV-lessons from the clinic. J Exp Med, 2017 214(2): p. 269–283. 28108590 10.1084/jem.20161846 PMC5294862

[60] Price S, , Natural history of autoimmune lymphoproliferative syndrome associated with FAS gene mutations. Blood, 2014 123(13): p. 1989–99. 24398331 10.1182/blood-2013-10-535393 PMC3968385

[61] Rao VK and Oliveira JB, How I treat autoimmune lymphoproliferative syndrome. Blood, 2011 118(22): p. 5741–51. 21885601 10.1182/blood-2011-07-325217 PMC3228494

[62] Cohen JI, , Characterization and treatment of chronic active Epstein-Barr virus disease: a 28-year experience in the United States. Blood, 2011 117(22): p. 5835–49. 21454450 10.1182/blood-2010-11-316745 PMC3112034

[63] Hadjadj J, , Progressive Multifocal Leukoencephalopathy in Primary Immunodeficiencies. J Clin Immunol, 2019 39(1): p. 55–64. 30552536 10.1007/s10875-018-0578-8

[64] Caminha I, , Using biomarkers to predict the presence of FAS mutations in patients with features of the autoimmune lymphoproliferative syndrome. J Allergy Clin Immunol, 2010 125(4): p. 946–949 e6. 20227752 10.1016/j.jaci.2009.12.983 PMC3412519

